# Resilience to Diabetic Retinopathy (RDR) Is Associated with a Pre-Retinopathy Transcriptional Program Induced by Diabetes

**DOI:** 10.3390/biom16040614

**Published:** 2026-04-21

**Authors:** Janani Rajasekar, Maria Paula Zappia, Maximilian A. McCann, Maxim V. Frolov, Andrius Kazlauskas

**Affiliations:** 1 Department of Ophthalmology and Visual Sciences, University of Illinois Chicago, Chicago, IL 60612, USA; janani@uic.edu (J.R.); mccann7@uic.edu (M.A.M.); 2 Department of Biochemistry and Molecular Genetics, University of Illinois Chicago, Chicago, IL 60612, USA; mpzappia@uic.edu (M.P.Z.); mfrolov@uic.edu (M.V.F.); 3 Department of Physiology and Biophysics, University of Illinois Chicago, Chicago, IL 60612, USA

**Keywords:** diabetic retinopathy, resilience to diabetic retinopathy, Müller cells, single-cell RNA sequencing

## Abstract

The purpose of this project was to define gene expression changes associated with the acquisition and loss of resilience to diabetic retinopathy (RDR) in individual retinal cell types. A non-immune form of type 1 diabetes mellitus (DM) was induced by injecting male *C57Bl6J* mice with streptozotocin. Single-cell RNA sequencing was performed on retinas from mice that experienced DM for 5 or 15 days, along with retinas from age-matched, non-DM mice. The resulting data sets were analyzed to identify DM-associated differentially expressed genes and pathway enrichments after each duration of DM. We observed that acquisition of RDR, previously shown to arise after 5 days of DM was linked to altered expression of genes in a subset of retinal cells, mainly Müller cells. Pathway analysis indicated enhancement of numerous modes of protection, including reinforced neurovascular and structural homeostasis through phagocytosis, integrin signaling, and interferon-mediated defense. After 15 days of DM, when we previously showed that RDR is waning this pro-protection surge in gene expression subsided. We conclude that a duration of DM that is too short to cause diabetic retinopathy (DR) nonetheless evoked a profound change in the gene expression profile within a subset of retinal cell types. The nature and timing of this molecular shift indicated that it was not the preamble to DM-related damage that eventually develops. Rather, DM engaged numerous defense programs within Müller cells. The temporal alignment between RDR and activation of Müller cell-based defense provides a molecular foundation for the retina’s transient ability to remain healthy in the face of DM.

## 1. Introduction

Evidence for the existence of endogenous defense mechanisms against disease can be inferred from the age-related incidence of pathology. For instance, the likelihood of developing cardiovascular disease is substantially lower before age 50 than after age 70 [1]. A well-recognized component of this intrinsic defense is the immune system, whose function progressively declines with age [2]. In addition to immune-based mechanisms, non-immune processes such as hormesis also contribute to resilience against pathology [3,4,5]. A deeper understanding of these endogenous defense systems will guide the development of therapeutic strategies that sustain health [6]. Diabetic retinopathy, which manifests only after a prolonged duration of diabetes [7,8,9,10,11], provides an ideal context in which to investigate endogenous defense mechanisms.

Diabetes mellitus (DM) is a major health issue. According to the International Diabetes Federation, 10.5% of the world’s population is diabetic. The U.S. Centers for Disease Control and Prevention (CDC) reports that the incidence of DM is increasing, attempts to reverse this trend have not been successful, and that there is no cure for DM. DM greatly increases an individual’s susceptibility to a plethora of health issues that compromise an individual’s quality of life. The vast number of people who currently have DM (860 million) and the anticipated increase in their number constitute a pressing need to address the health issues of people with DM.

Diabetic retinopathy (DR) is the most common microvascular complication of DM and the leading cause of blindness among working-age individuals [12]. DR eventually develops in the vast majority of patients with DM [13,14] and is the most-feared diabetic complication, perhaps because loss of vision profoundly impacts an individual’s quality of life.

The long delay between the onset of DM and the manifestation of DR [7,8,9,10,11] indicates that the retina is resistant to DM-induced damage. The retina remains healthy after 10 years of DM in over 80% of patients with Type 2 DM (T2DM) [15]. Similarly, it takes patients with Type 1 DM (T1DM) an average of 21.3 years to develop vision-threating proliferative diabetic retinopathy (PDR) [16]. The translational relevance of resilience to DR (RDR) and the existence of animal models of RDR [17,18] make this component of DR pathogenesis a fertile opportunity [19,20].

The RDR phenomenon has been investigated in patients with T1DM who are extraordinarily resistant to DR (participants of the Joslin 50-Year Medalist Study [21]). They have a high level of retinol binding protein 3 (RBP3) in their retinas [22]. In a different population of patients, which included individuals with either T1DM or T2DM, an elevated level of RBP3 in the vitreous was associated with less severe DR and a reduced risk of developing PDR [23].

Investigation of RDR in experimental animals has revealed that cells within retinal vessels of both T1DM and T2DM mice are resistant to oxidative stress-induced death during RDR [24]. As RDR wanes and DR develops, the cells lose resistance and become more vulnerable to insult-induced death [24]. The underlying mechanism of RDR was investigated in primary human retinal endothelial cells and found to involve hyperglycemia-induced mitochondrial adaptation (HIMA), which entails a matched increase in the clearance of dysfunctional mitochondria (mitophagy) and biogenesis of new mitochondria [19,25]. Curiously, the mechanism of RDR in retinal pericytes is different from the HIMA-based mechanism operating in retinal endothelial cells [25]. While gene expression changes associated with DR have been reported [26,27,28], little is known about gene expression changes within the neural retina that occur during the RDR period.

Herein, we applied scRNA-Seq to the standard streptozotocin (STZ) murine model of T1DM [29] to investigate the transcriptional changes that occur as the retina acquires and then loses RDR. We discovered that RDR was temporally aligned with transcriptional activation of Müller cell-based defense programs, which dissipated as RDR deteriorated.

## 2. Materials and Methods

### 2.1. Experimental Animals

Diabetes was induced in eight-week-old male C57BL/6J mice (Jax #000664, Jackson Laboratory, Bar Harbor, ME, USA) by administering five consecutive daily intraperitoneal injections of streptozotocin (60 mg/kg/day) (S0130, Sigma-Aldrich, Burlington, MA, USA) in 47 mM sodium citrate (pH 4.5). Age-matched non-diabetic (non-DM) control mice received an equivalent volume of citrate buffer. Fasting blood glucose levels were measured two days after the final streptozotocin dose. Mice that had a fasting blood glucose level exceeding 250 mg/dL at this time point, which they maintained for 5 days or 15 days, were considered diabetic. Body weight and fasting blood glucose were monitored regularly throughout the diabetes duration. Mice were sacrificed the day after the designated time points for further analysis. The animals were housed in group cages under pathogen-free conditions, maintained on a 12 h light/dark cycle, with ad libitum access to food and water. Euthanasia was performed using CO_2_ asphyxiation, followed by immediate enucleation and processing of the eyes. All animal procedures were approved by the Office of Animal Care and Institutional Biosafety at the University of Illinois Chicago.

### 2.2. Sample Preparation

Eyeballs were enucleated, and the retinas were dissected and prepared for single-cell RNA sequencing (scRNA-Seq) following the 10× Genomics guidelines (#1000414, CG000553, 10× Genomics, Pleasanton, CA, USA). In brief, the retinal tissue, separated from the choroid and retinal pigment epithelium layer, was fixed in fixation buffer for 24 h. Following fixation, the retinal tissue was dissociated into a single-cell suspension using dissociation buffer for 20 min. The resulting suspension was filtered through a 40 μm strainer to ensure a single-cell population. The cells were then resuspended in quenching buffer for storage at −80°C. Retinal samples for the 5-day DM, 5-day non-DM, 15-day DM, and 15-day non-DM conditions were prepared from a total of three mice for each condition (*n* = 3). Sample quality was assessed by staining cells with Acridine Orange/Propidium Iodide dye and visualized using the LUNA-FX7™ Automated Cell Counter (Logos biosystems). The samples were processed following the user Guide (CG000691, 10x Genomics) for Chromium Fixed RNA Profiling assay using the Chromium X (10x Genomics). Briefly, between 4,000 and 10,000 cells from the retinal single-cell suspension were captured for each condition after hybridization with Mouse WTA Probes. Libraries were made, checked using High Sensitivity D5000 ScreenTape and the TapeStation 4150 (Agilent), quantified using KAPA library quantification kit, and sequenced into a 10B lane NovaSeq X Plus (Illumina). The fastq files were demultiplexed and aligned to Mus musculus genome assembly GRCm38 (mm10/ Ensembl98) using cellranger-7.0.1.

### 2.3. scRNA-Seq Data Analysis

Single-cell RNA-Seq data from the four cohorts described above were processed in Seurat (v5.3.0). Datasets from all four cohorts were merged for initial preprocessing. Cells were filtered to exclude low-quality populations based on the following criteria: <200 or >8,000 detected genes, <500 or >25,000 total counts, or mitochondrial transcript fraction ≥ 5%. Dimensionality reduction was performed by principal component analysis, and the first 20 principal components were used for graph-based clustering at a resolution of 0.6.

### 2.4. Differential Gene Expression

Differential gene expression was assessed at the cell-type level for the 5-day and 15-day DM time points using the Wilcoxon rank-sum test implemented in Seurat’s FindMarkers. For each non-DM/DM pairwise comparison, cells were subset to the relevant cell type and DM duration prior to testing. Genes were considered differentially expressed if they met all the following thresholds: min.pct = 0.1, logfc.threshold = log(1.5), and adjusted *p*-value < 0.05.

### 2.5. Pathway Analysis

Functional enrichment was evaluated using Gene Set Enrichment Analysis (GSEA), Gene Ontology Biological Process (GOBP) enrichment, and Ingenuity Pathway Analysis (IPA). GSEA and GOBP were performed in R (version 4.3.2) using gene sets from MSigDB (gsea-msigdb.org, accessed on 17 September 2025). Visualization of enrichment results was carried out with ggplot2 (version 3.5.2). Differentially expressed genes (DEGs) from pairwise comparisons between DM and non-DM mice at each time point were used as input. For IPA (Qiagen; https://www.qiagenbioinformatics.com/products/ingenuity-pathway-analysis/, accessed on 17 September 2025), DEGs with adjusted *p* value < 0.05 from the same comparisons were analyzed.

### 2.6. qRT-PCR

Retina from non-DM and DM mice were collected at the 5-day time point. Total RNA was extracted from whole retina using Qiagen kit and cDNA was synthesized with HighCapacity cDNA Reverse Transcriptase kit (ThermoFisher, Waltham, MA, USA) following standard protocols. Gene expression was assessed by qPCR on QuantStudio 7 Flex system using Fast SYBR Green Master Mix (Applied Biosystems, Waltham, MA, USA). Primer sequences used in this study are listed below.
**Gene****Forward Primer****Reverse Primer***Actb*5′-GAG AGG TAT CCT GAC CCT GAA-3′5′-TGA AGG TCT CAA ACA TGA TCT GG-3′*Selenbp1*5′-TCATGGTCAGCACTGAATGG-3′5′-CTGCCAGTCCCACACAAATA-3′*Bcl2l10*5′-ACCTACCTGTGAACCCTCTAA-3′5′-CCATGTAGCATGCAGGTAGAA-3′*Septin4*5′-CCTGAAGTGGACCGAAAGAAA-3′5′-TCACAGTCTGGGAACTGATAGA-3′*Nup50*5′-AGCGGTGAGGTTGCTAATAC-3′5′-GGGATTAGACAGGAACCATGAC-3′*Glmp*5′-CTCGTGGAAACCATTCCCTATT-3′5′-GAAGACGGCAGGTGCATATT-3′*Ddit4*5′-CAGGAAGTCTCTAGGTTGTATGC-3′5′-GAAGCCTGTGTGACTCCTAAG-3′

### 2.7. Statistical Analyses

For the scRNA-Seq analyses, the Wilcoxon test was applied. For comparisons between two groups, data were analyzed using a two-tailed Student’s *t*-test. Statistical significance was defined as *p* < 0.05 (*), *p* < 0.01 (**), *p* < 0.001 (***) and *p* < 0.0001 (****).

## 3. Results

### 3.1. Profiling Changes in the Molecular Landscape of the Neural Retina That Are Associated with the Acquisition of RDR and Its Deterioration

**Figure 1:**
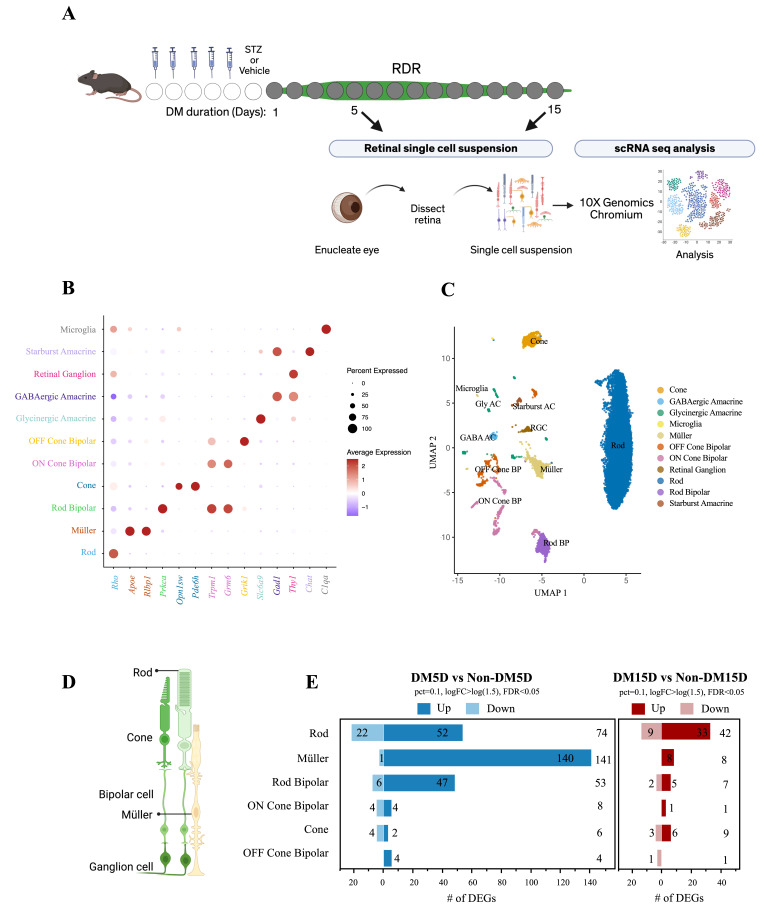
Acquisition of RDR was associated with a wave of transcriptional activity that waned as RDR deteriorated. (**A**) Schematic representation of the experimental workflow. DM was induced with STZ (streptozotocin) as described in the Section 2. There were 4 cohorts of mice: DM for 5 or 15 days, along with age-matched non-DM control mice. In this model, the duration of DM after which mice have acquired (5 days) and begin to lose RDR (15 days) has been previously established [24]. Low-level RDR is also detectable after 28 days of DM; however, at this duration of DM the vasculature of some of the DM mice show increased vulnerability to oxidative stress-induced death as compared with the non-DM mice [24]. (**B**) Dot plot illustrating the expression of canonical marker genes (x axis) that used to identify major retinal cell types (y axis). The color and size of the symbols indicate average expression and percentage of expressing cells, respectively. (**C**) UMAP plot showing cell type annotations for each cluster. (**D**) Graphical representation of retinal organization showing neural cells (rods, cones, bipolar cells, and ganglion cells) together with supporting Müller glial cells. (**E**) The number of upregulated and downregulated differentially expressed genes (DEGs) for individual cell types at DM5D and DM15D time points. The total # of DEGs for each cell type is indicated at the far-right side of the box. UMAP: Uniform manifold approximation and projection.

We investigated the DM-induced, cell type-specific changes in gene expression that were associated with acquisition of RDR and its subsequent erosion. To this end, we performed single-cell transcriptomic profiling of the retina after 5 days of DM, a duration that induces RDR, and after 15 days of DM, when RDR is deteriorating [24,30,31]. The non-immune-mediated type 1 DM murine model was used in these studies because the duration of DM is known with greater precision in this model than with other models such as the *db/db* genetic model of type 2 DM [18,29,32]. To induce DM, STZ was injected on 5 consecutive days; fasting blood glucose was measured daily from day 7 until sacrifice (Figure S1). Injection of the STZ vehicle did not induce DM (Figure S1). In these experiments, there was a total of four cohorts: DM for 5 (DM5D) or 15 days (DM15D), and the age-matched non-DM controls. A schematic overview of the experimental workflow is shown in Figure 1A.

Sequencing data from all four cohorts were combined for the initial stages of data analysis. A total of 23,901 cells were sequenced, with a median of 2170 genes detected per cell. After quality control and filtering using the Seurat pipeline, 20,759 high-quality cells were retained for downstream analysis. Unsupervised clustering identified 28 transcriptionally distinct clusters (Figure S2A).

To organize the dataset according to retinal cell types, the 28 transcriptionally distinct clusters were consolidated into 11 groups based on the expression of genes that encode canonical cell-type markers (Figure 1B). For example, clusters expressing Rho were annotated as rods, whereas those expressing Opn1sw as cones (Figure 1B,C). Clusters lacking clear cell type-specific gene signatures (clusters 8, 13, and 24 in Figure S2A) were excluded from further analysis. Some cell types (e.g., glycinergic amacrine (Gly AC)) existed in multiple locations within the UMAP plot (Figure 1B), indicating that they had more than one transcriptional profile. There was a similar profile of cell types from each of the four cohorts of mice (Figure S2B). As reported by others, we did not detect cell types that constitute less than 1% of the adult mouse retina, such as astrocytes, horizontal cells, endothelial cells, and pericytes [26,27]. At each of the two durations of DM, the DM and non-DM data sets were compared to identify the DM-induced differentially expressed genes (DEGs) that were statistically significant (FDR < 0.05, pct = 0.1, and logFC > log(1.5).

To complement the scRNA-Seq data, we analyzed the effect of 5 days of DM on gene expression in the total retina by qRT-PCR (Figure S3). We focused on the DEGs identified in rods because this cell type is the most abundant in the retina (approximately 65% [26,27]) and because this duration of DM elicited the most pronounced transcriptional response (Figure 1). While the scRNA-Seq and PCR results aligned for some genes (*Nup50*, *Ddit4*), this was not always the case. One likely explanation for the dissonance is that the expression of certain genes was altered by DM in rods but not in other retinal cell types. Because qRT-PCR was performed using RNA isolated from the total retina, the presence of other cell types may have diluted rod-specific changes, thereby masking differential expression that was detectable by scRNA-Seq, where gene expression was evaluated at the resolution of individual cell types.

### 3.2. RDR Induced the Greatest Response in Müller Cells

While all cell types within the retina presumably experienced DM (because it is systemic), expression of genes changed in only a subset of retinal cell types (Table 1, Figure 1D,E). No DEGs were detected in the least abundant cell types, which may be at least in part due to the relatively shallow and non-comprehensive nature of scRNA-Seq [33,34,35]. However, among the cell types that harbored DEGs, their relative abundance did not correlate with the number of genes altered in response to DM (Figure 1D,E, and Table 1). Five days of DM altered the expression of 1.9-fold more genes in Müller cells compared to rods, even though there were 15 times more rods than Müller cells (Table 1 and Figure 1E). Moreover, the DEGs/cell ratio was dissimilar in cell types that were equally abundant (Müller and rod bipolar; Table 2). These data indicate that there was a cell type-specific response to DM, which is consistent with previous publications [27,36], and that the response in Müller cells was greater than in any of the other cell types.

### 3.3. RDR Was Associated with a Wave of Transcriptional Activity That Waned by the Onset of DR

In the context of DR, which intensifies as the duration of DM is prolonged [8], the expectation is that the number of DM-induced DEGs will increase as the duration of DM is extended. These experiments were designed to assess the effect of RDR, instead of DR, on gene expression. This is an unpreceded line of investigation, and therefore without expectation. We observed that RDR was associated with a wave of transcriptional activity: the peak of activity temporally aligned with acquisition of RDR (Figure 1E). Extending the duration of DM reduce the number of DEGs. After 15 days of DM, the number of DEGs in rods, Müller cells, and rod bipolar cells dropped to 57%, 6%, and 13% of the levels observed at 5 days, respectively (Figure 1E). We conclude that the response to DM is cell type-specific and that DM triggered a wave of transcriptional activity during RDR, which largely disappeared as RDR waned [24].

### 3.4. The Acquisition of RDR Was Associated with the Activation of Endogenous Defense Systems Within Müller Cells

Müller cells defend the retina through numerous mechanisms including regulation of synaptic activity in the inner retina via neurotransmitter uptake and exchange, and phagocytosis of photoreceptor outer segment disks to support photoreceptor renewal. They also control the blood–retina barrier and local blood flow, thereby maintaining neurovascular coupling between the neural and vascular compartments [37,38,39,40,41]. Because Müller cells span the full thickness of the retina and physically interact with all retinal cell types, they also contribute to mechanical homeostasis [42,43].

**Figure 2:**
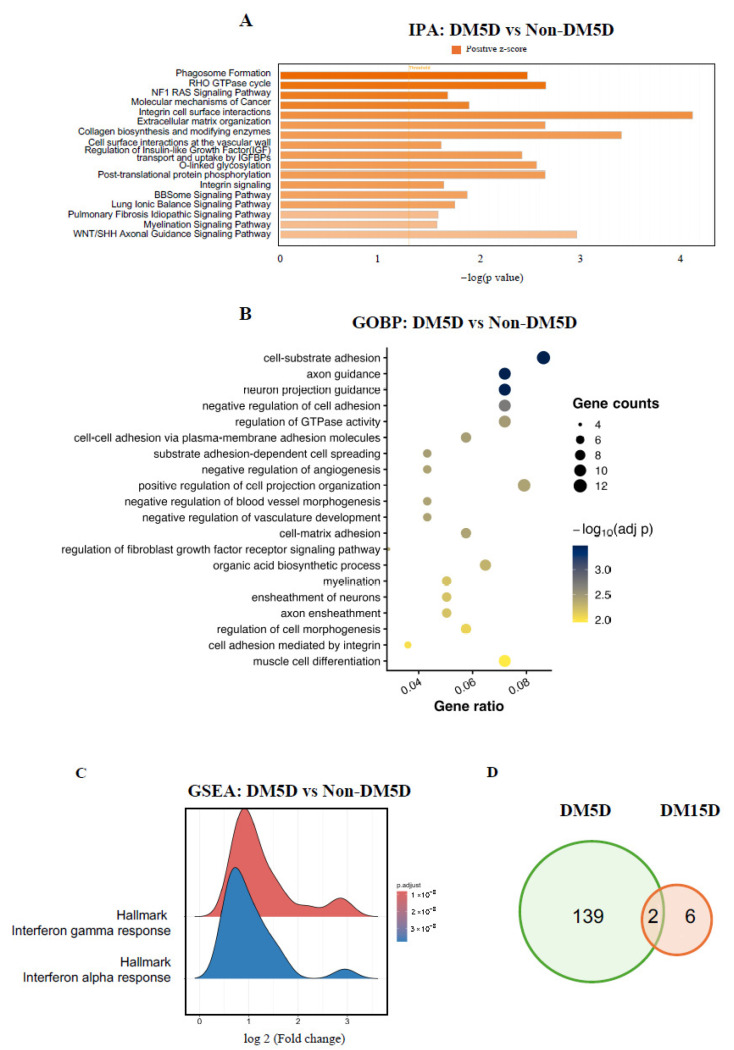
Pathway analysis revealed a protective response of Müller glial cells during RDR. (**A**,**B**) Pathway analysis of the 141 DEGs (DM5D versus Non-DM5D) in Müller cells, was performed using Ingenuity Pathway Analysis (IPA; (**A**) and Gene Ontology biological processes (GO BP; (**B**)). (**C**) Gene Set Enrichment Analysis (GSEA). (**D**) Venn diagram depicting the number of unique and shared DEGs after 5 and 15 days of DM.

The gene expression changes that coincided with acquisition of RDR in Müller cells engaged these core protective functions (Figure 2). IPA identified strong activation of “Phagosome Formation,” “Integrin Cell Surface Formation,” “Integrin Signaling,” “Extracellular Matrix Formation,” and “Cell-Surface Interactions at the Vascular Wall” (Figure 2A). GOBP analysis confirmed this concept; “cell–substrate adhesion” was among the most upregulated pathways (Figure 2B). Finally, GSEA revealed enrichment of “Hallmark Interferon γ Response” and “Hallmark Interferon α Response” (Figure 2C). Acute activation of these pathways engages the innate immune response in ways that are protective [44,45,46,47]. Together, these data show that acquisition of RDR was associated with transcriptional changes that enhanced many of the processes by which Müller cells protect the retina from adversity.

Like RDR itself, the transcriptional changes associated with it were transient (Figure 2D). Pathway analysis of the eight DEGs present after 15 days of DM did not identify any pathways. Thus, deterioration of RDR was associated with a reversal of transcriptional changes that occurred at its onset, as opposed to a novel set of gene expression changes. We conclude that RDR engaged multiple protection-related responses in Müller cells that subsided as RDR deteriorated.

### 3.5. The Transcriptional Response to RDR in Rods

**Figure 3:**
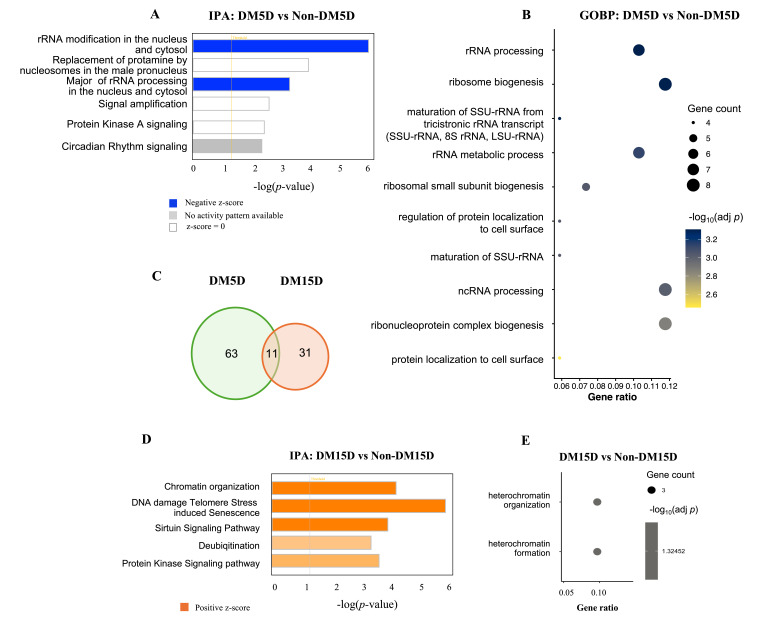
The transcriptional response of rods to RDR. (**A**,**B**) Pathway analysis of differentially expressed genes (DEGs) between DM5D and Non-DM5D using IPA (**A**) and GOBP (**B**). (**C**) Venn diagram depicting the number of unique and shared DEGs after 5 and 15 days of DM. (**D**,**E**) Pathway analysis of DEGs between DM15D and Non-DM15D using IPA (**D**) and GOBP (**E**).

Two other retinal cell types also underwent robust RDR-associated changes in gene expression (Figure 1E); however, in both cases the changes were aligned with damage instead of protection. The transcriptional response of rods was a suppression of pathways related to “ribosomal biogenesis”, rRNA modification”, and “rRNA processing” (Figure 3A,B). Rods are particularly dependent on these processes because of the continual demand for large amounts of the proteins (e.g., rhodopsin) necessary for phototransduction and maintenance of their outer segment discs [48]. Consequently, acquisition of RDR appeared to result in dysfunction of key components of the cells’ infrastructure necessary to translate mRNA [49,50,51]. As RDR eroded, this transcriptional profile resolved and was replaced by activation of “chromatin organization” and “heterochromatin organization” pathways (Figure 3C–E). Such large-scale remodeling of the transcriptional landscape may be responsible for the restoration of homeostatic ribosomal biogenesis. Nonetheless, evidence of persistent stress remained at the 15-day DM time point, as indicated by activation of the “DNA damage, Telomerase, and Stress-induced Senescence” pathway. While relatively modest, this transcriptional signature of dysfunction aligns with the onset of ERG dysfunction at this duration of DM [30,31].

### 3.6. Rod Bipolar Cells Underwent Transient Transcriptional Changes That Indicated Dysfunction During RDR

**Figure 4:**
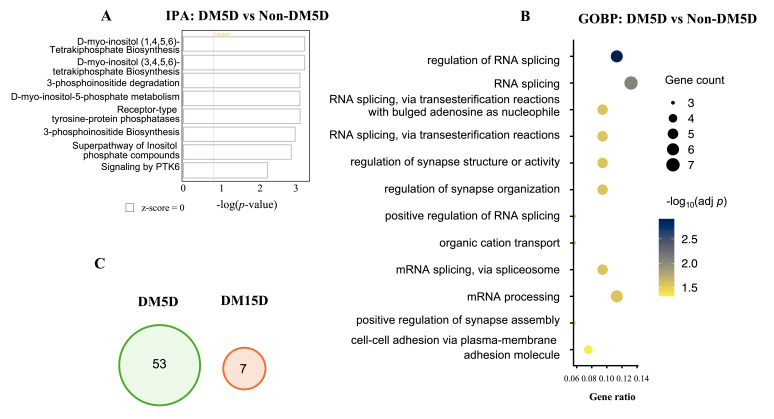
Transcriptional signatures of rod bipolar cells to RDR. (**A**,**B**) Pathway analysis of differentially expressed genes (DEGs) between DM5D and Non-DM5D in rod bipolar cells using IPA (**A**) and GOBP (**B**). (**C**) Venn diagram depicting the number of unique DEGs after 5 and 15 days of DM.

Rod bipolar cells synapse with rods to integrate and transmit their signals to other retinal neurons, such as amacrine cells [52]. Rod bipolar cells splice RNA to generate protein isoforms that are essential for their function. For instance, these cells generate an isoform of retinal Purkinje cell protein-2, which enables the hyperpolarization of the dark membrane potential and speeds up the light response [53]. Acquisition of RDR was associated with suppression of “regulation of RNA splicing” and “RNA splicing” pathways in GOBP, while IPA did not indicate corresponding pathway alterations and showed no clear directional change (Figure 4A,B). As the duration of DM was prolonged, this gene signature vanished and was replaced with altered expression of 7 genes, which were not diagnostic of any specific pathways (Figure 4C). We conclude that, like rods, the transcriptional response to the acquisition of RDR in rod bipolar cells was deleterious and resolved as the duration of DM was prolonged.

## 4. Discussion

Herein we report that DM triggered a wave of transcriptional changes that temporally aligned with the previously reported appearance and subsequent erosion of RDR [24]. Even though DM is systemic, the response of retinal cell types was non-uniform. The transcriptional response in Müller cells was greatest, both in the number of genes and the magnitude of the change in expression. This early response to DM engaged numerous defense-related pathways and thereby provides a molecular basis for the ability of the retina to initially remain healthy in the face of DM [18,30,31].

Consistent with these observations, other groups have also reported increased expression of protection-related genes such as *Zfp36* [54,55], which encodes an RNA binding protein that promotes the degradation of proinflammatory RNAs [56]. Similarly, *Agtpbp1* (also known as *Nna1*), which encodes a metallocarboxypeptidase that mediates protein deglutamylation of tubulin and non-tubulin target proteins [57,58,59], is expressed in Müller cells and protects diabetic rats from DR [60]. While these investigators did not consider the contribution of these genes to RDR, the variety of protection-related genes expressed by Müller cells suggests that RDR is transcriptionally complex.

Since DM is a metabolic dysfunction, we anticipated that metabolism-related genes would be affected, but none were detected. A plausible explanation is that not all metabolic perturbations require a change in gene expression [61,62].

It is likely that the transcriptional changes reported herein underestimate RDR-associated gene expression changes and the cell types that are involved. A qRT-PCR-based approach detected increased expression of anti-oxidative stress genes in the RDR retina [24], a signature that was not present in our scRNA-Seq dataset. The relatively shallow and non-comprehensive nature of scRNA-Seq [33,34,35] is a possible explanation. Furthermore, not all retinal cell types were present in our scRNA-Seq (Figure 1C). Notable absent are vascular cell types, which are known to undergo RDR [19,24,25]. Alternative approaches are necessary to identify the RDR-associated transcriptional changes in endothelial cells and pericytes.

It is conceivable that STZ, which was used to induce DM, contributed to the RDR-associated changes in gene expression. However, several lines of evidence argue against this possibility. First, STZ enters cells via glucose transporter 2 (GLUT2), which is not expressed by any of the retinal cell types [63]. Furthermore, concentrations of STZ that are cytotoxic to GLUT2-expressing cells are non-cytotoxic to cells lacking GLUT2 [64]. Second, the DM5D cohort was harvested well after STZ would have been degraded. STZ has a half-life of approximately 15–20 min at neutral pH in peritoneal fluid [65,66,67], and DM5D mice were sacrificed 8 days after the final STZ injection (Figure S1), corresponding to 576–768 half-lives. Finally, RDR-associated transcriptional changes are also observed in a genetic model of RDR/DR (*db/db* mice) that does not involve STZ exposure [24]. Taken together, we favor the interpretation that persistent hyperglycemia instead of the brief exposure to STZ was the primary driver of the altered gene expression program.

The priorities of this project did not include assessing fluctuation in the level of hyperglycemia, which increases the risk of DR in patients [68]. While this aspect of clinical DM is present in the mouse model used in these studies [69], we measured blood glucose only once/day and therefore did not determine the extent of daily fluctuation of hyperglycemia in our cohorts of mice.

Although our scRNA-Seq analysis identified a Müller cell-centric protective signature, it is unlikely that Müller glia are the sole mediators of retinal defense during RDR. Other retinal cell types, particularly endothelial cells within the retinal vasculature, have been shown to protect the retina from a variety of insults including DM [70,71,72]. Emerging evidence suggests that this endothelial cell-based protection involves enhanced mitochondrial flux, characterized by a coordinated increase in clearance of damaged mitochondria (mitophagy) and mitochondrial biogenesis [19,25]. This dynamic remodeling of the mitochondrial network supports sustained bioenergetic capacity and limits oxidative stress under prolonged hyperglycemic conditions. This mitochondrial-based defense used by endothelial cells was not detected in Müller cells. Together these findings suggest that multiple cell types act via distinct mechanisms to enable the retina to remain healthy in the face of persistent hyperglycemia.

The translation benefit of investigating RDR is substantial. Advancing our understanding of what induces RDR and why it deteriorates will enable the development of novel approaches to prevent at-risk patients from developing DR. Currently, 26% of patients with DM have DR, indicating that available prophylactic approaches (e.g., curbing blood sugar) are not effective for over 1/4 of this patient population [73]. RDR-based therapeutics will extend the duration of tolerance to hyperglycemia and thereby provide a new therapeutic approach to prevent DR.

Limitations of the study include that the approach was primarily bioinformatics and the paucity of cell type-specific in vitro RDR assays with which to validate the results.

## 5. Conclusions

A duration of DM that was too short to cause overt DR nonetheless evoked a profound change in the gene expression profile within a subset of retinal cell types. The nature and timing of this molecular shift indicated that it was not the preamble to DM-related damage that eventually develops. Rather, DM engaged numerous defense programs within Müller cells. These observations provide a molecular foundation for the retina’s ability to initially remain healthy in the face of DM.

## Figures and Tables

**Table 1:** Average number of each cell type that was analyzed.

Cell Type	Average Number of Cells Sequenced *	Percentage of the Total (%)
Rod	3420	74.3
Müller	224	4.8
Rod Bipolar	223	4.9
Cone	187	4.1
ON Cone Bipolar	194	4.1
OFF Cone Bipolar	161	3.5
Glycinergic Amacrine	107	2.4
GABAergic Amacrine	30	0.7
Retinal Ganglion	25	0.6
Starburst Amacrine	25	0.5
Microglia	8	0.2

* These values are the average of all 4 cohorts of mice.

**Table 2:** Number of DEGs/Cell after 5 and 15 days of DM.

Cell Type	Number of DEGs/Cell DM5D	Number of DEGs/Cell DM15D
Rod	21.6	12.3
Müller	629.5	35.7
Rod Bipolar	237.7	31.4
Cone	32.1	48.1
ON Cone Bipolar	41.2	5.2
OFF Cone Bipolar	24.8	6.2

## Data Availability

All single-cell sequencing data for this study will be deposited in NCBI’s Gene Expression Omnibus upon acceptance for publication.

## Supplementary Materials

The following supporting information can be downloaded at: https://www.mdpi.com/article/10.3390/biom16040614/s1, Figure S1: The level of fasting blood glucose of the mice. Eight-week-old male C57BL6J mice were injected daily for 5 consecutive days with STZ (60 mg/kg dissolved in citrate buffer, pH= 4.5) or vehicle (citrate buffer, pH=4.5). They were rested for two days and then the fasting blood glucose was monitored daily until the end of the experiment. Each point in the graph is the average level of fasting blood glucose of the 3 mice in each cohort; Figure S2: Single-cell transcriptomic analysis identified 28 transcriptionally distinct clusters; Figure S3: qRT-PCR analysis of expression in the retina from mice that were DM for 5 days along with the age-matched non-DM control mice. RNA isolated from the retina was analyzed by qRT-PCR using primers directed to the indicated genes.; *Actb* was used as the housekeeping gene. "Relative expression": normalized expression (ratio of gene of interest/housekeeping gene) of a given gene in DM versus non-DM. Each point indicates the normalized expression in an individual mouse. The unpaired *t*-test was used to assess statistical significance between non-DM and DM pairs; *p* < 0.05 (*), and *p* < 0.001 (***); Table S1: Differential gene expression in Rod at DM5D, highlighting the most significantly upregulated and downregulated genes with an average log fold change ≥ 0.5.

## Abbreviations

The following abbreviations are used in this manuscript:

DM	Diabetes Mellitus
DR	Diabetic Retinopathy
DEGs	Differentially Expressed Genes
GOBP	Gene Ontology Biological Process
GSEA	Gene Set Enrichment Analysis
GLUT2	Glucose Transporter 2
HIMA	Hyperglycemia induced Mitochondrial Adaptation
IPA	Ingenuity Pathway Analysis
PDR	Proliferative Diabetic Retinopathy
RDR	Resilience to Diabetic Retinopathy
RBP3	Retinol Binding Protein 3
scRNA seq	Single cell RNA seq
STZ	Streptozotocin
T1DM	Type 1 Diabetes Mellitus
T2DM	Type 2 Diabetes Mellitus
