# Supplementary material for: Resilience to Diabetic Retinopathy (RDR) Is Associated with a Pre-Retinopathy Transcriptional Program Induced by Diabetes
Source: Biomolecules. 2026 Apr 21;16(4):614. doi: 10.3390/biom16040614 (PMC13114160; doi:10.3390/biom16040614)
Supplement: Supplementary file 1 [file biomolecules-16-00614-s001.zip › biomolecules-4226390-supplementary.pdf]

Figure S1

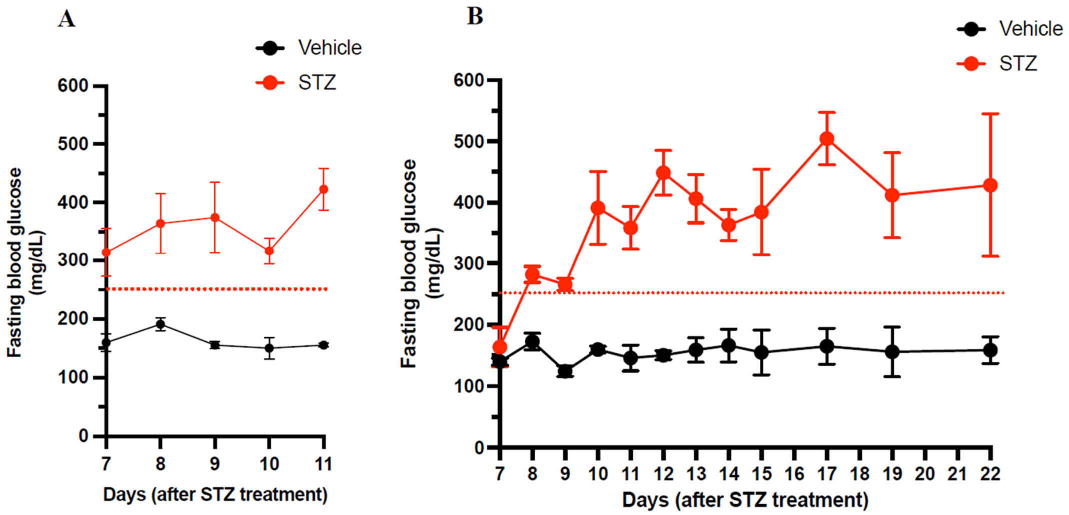

Figure S1. The level of fasting blood glucose of the mice. Eight-week-old male C57BL6J mice were injected daily for 5 consecutive days with STZ (60 mg/kg dissolved in citrate buffer, pH= 4.5) or vehicle (citrate buffer, pH=4.5). They were rested for two days and then the fasting blood glucose was monitored daily until the end of the experiment. Each point in the graph is the average level of fasting blood glucose of the 3 mice in each cohort. Panels A and B: 5 and 15 day cohorts, respectively. Diabetes was defined as a fasting blood glucose >250 mg/dl; shown as a dotted red line.

Figure S2

A

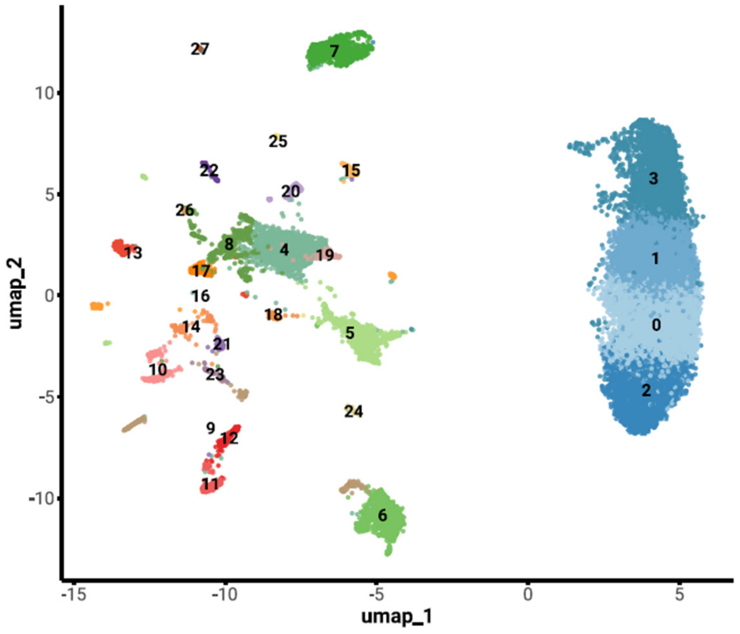

B

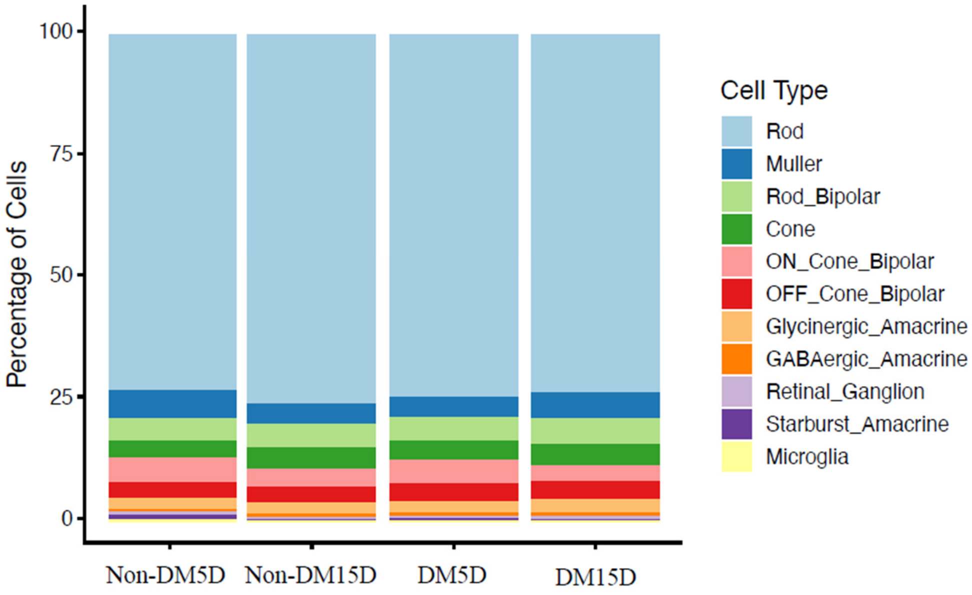

Figure S2. Single-cell transcriptomic analysis identified 28 transcriptionally distinct clusters. (A) UMAP showing all 28 transcriptional clusters identified before filtering for cell type-specific genes. (B) The percentage of each cell type in each of the 4 cohorts.

Figure S3

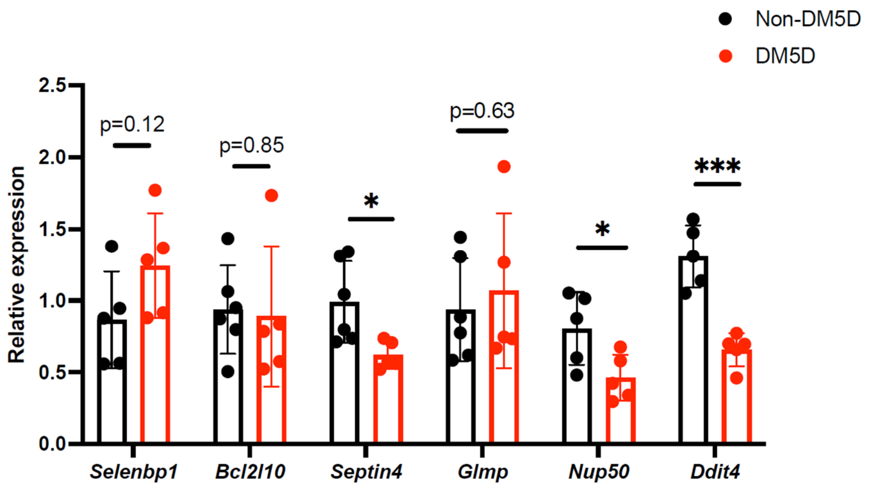

Figure S3. qRT-PCR analysis of expression in the retina from mice that were DM for 5 days along with the age-matched non-DM control mice. RNA isolated from the retina was analyzed by qRT-PCR using primers directed to the indicated genes.; *Actb* was used as the housekeeping gene. "Relative expression": normalized expression (ratio of gene of interest/housekeeping gene) of a given gene in DM versus non-DM. Each point indicates the normalized expression in an individual mouse. The unpaired t-test was used to assess statistical significance between non-DM and DM pairs; p < 0.05 (*), and p < 0.001 (***).

Table S1: Differential gene expression in Rod at DM5D, highlighting the most significantly upregulated and downregulated genes with an average log fold change ≥ 0.5.

Gene	p_val	avg_log2FC	pct.1	pct.2	p_val_adj
H4c3	5.71638678211799e-19	0.988	0.193	0.118	1.08948615680387e-14
Fcer1g	1.30467100450239e-14	0.958	0.118	0.066	2.4865724674811e-10
Selenbp1	1.8635485534975e-24	0.857	0.185	0.101	3.55173718811089e-20
Plekhg4	1.75798790598515e-13	0.726	0.17	0.11	3.35054915001709e-09
Bcl2l10	3.13441021292627e-16	0.72	0.223	0.147	5.97387242481617e-12
H1-4	1.47326684174625e-34	0.714	0.452	0.322	2.80789927368418e-30
Abhd14b	1.12638389083853e-14	0.682	0.157	0.095	2.14677505754915e-10
Septin4	5.57544494124288e-09	0.627	0.107	0.068	0.00010626
Pkp4	1.44830602393106e-06	0.607	0.102	0.07	0.02760327
Slc43a2	6.3777998211173e-14	-0.91	0.111	0.173	1.21554486790675e-09
Glmp	1.66882302523203e-10	-0.727	0.137	0.191	3.18060980378972e-06
Nup50	5.9332078592359e-07	-0.663	0.125	0.165	0.0113081
Ddit4	9.45333039625065e-26	-0.602	0.42	0.51	1.80171024022141e-21
Heatr1	2.47650954695205e-06	-0.559	0.138	0.178	0.0471998
Nol10	1.48939274076837e-09	-0.545	0.104	0.153	2.83863362463043e-05
Cpsf4	4.03526631558756e-07	-0.539	0.144	0.187	0.00769081

Polr3d	2.97549519986721e-08	-0.537	0.113	0.158	0.0005671
Arsg	1.22536056773631e-11	-0.512	0.187	0.25	2.33541470604863e-07
Tbl3	4.11412830635686e-07	-0.51	0.115	0.155	0.00784112
Krr1	1.80980489678134e-07	-0.508	0.242	0.29	0.00344931
